# MedML: Fusing medical knowledge and machine learning models for early pediatric COVID-19 hospitalization and severity prediction

**DOI:** 10.1016/j.isci.2022.104970

**Published:** 2022-08-17

**Authors:** Junyi Gao, Chaoqi Yang, Joerg Heintz, Scott Barrows, Elise Albers, Mary Stapel, Sara Warfield, Adam Cross, Jimeng Sun, N3C consortium

**Affiliations:** 1 University of Illinois Urbana Champaign, Champaign, IL, USA; 2 University of Illinois, College of Medicine Peoria, Department of Research Services, Peoria, IL, USA; 3 OSF HealthCare, Peoria, IL, USA; 4 Center of Excellence for Suicide Prevention, Department of Veterans Affairs, Department of Veterans Affairs, Canandaigua, NY, USA; 5 University of Illinois, College of Medicine Peoria, Department of Pediatrics, Peoria, IL, USA

**Keywords:** Respiratory medicine, Pediatrics, Artificial intelligence, Artificial intelligence applications

## Abstract

The COVID-19 pandemic has caused devastating economic and social disruption. This has led to a nationwide call for models to predict hospitalization and severe illness in patients with COVID-19 to inform the distribution of limited healthcare resources. To address this challenge, we propose a machine learning model, MedML, to conduct the hospitalization and severity prediction for the pediatric population using electronic health records. MedML extracts the most predictive features based on medical knowledge and propensity scores from over 6 million medical concepts and incorporates the inter-feature relationships in medical knowledge graphs via graph neural networks. We evaluate MedML on the National Cohort Collaborative (N3C) dataset. MedML achieves up to a 7% higher AUROC and 14% higher AUPRC compared to the best baseline machine learning models. MedML is a new machine learnig framework to incorporate clinical domain knowledge and is more predictive and explainable than current data-driven methods.

## Highlights

• MedML extracts the most predictive features from over 6 million medical concepts
• MedML fuses medical knowledge into machine learning models via graph neural networks
• MedML outperforms other methods on pediatric COVID-19 predictions using N3C dataset

## Graphical abstract

**Figure:**
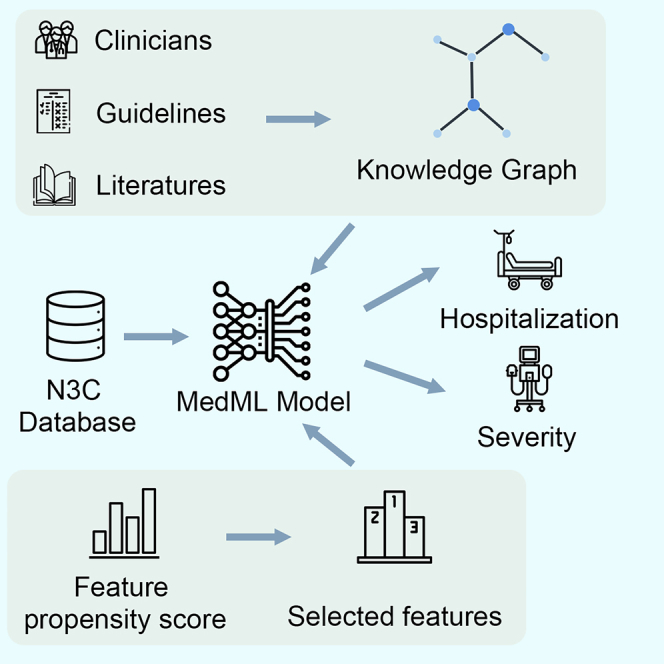

## Introduction

The COVID-19 pandemic needs no introduction. With over 81.5 million confirmed cases and 995,000 deaths by May 5, 2022, the mounting burden of this disease on global health has impacted communities on every continent (Centers for Disease Control and Prevention, 2022). While mortality is dramatically higher in adults than in children, the recent prevalence of the Omicron variant has sparked a surge in pediatric COVID-19 hospitalizations worldwide (Belay and Godfred-Cato, 2022; Mowery, 2022). In January 2022, an average of 1,015 pediatric inpatients were reported by each state, far surpassing last winter’s daily record of roughly 400 per state (American Academy of Pediatrics, 2022). While many of these patients are asymptomatic, others present a wide array of symptomologies, including bronchiolitis, pneumonia, and laryngotracheobronchitis (croup). The continued rise in case numbers, variable semiology, and healthcare personnel shortages across the country underscores the urgent need to accurately predict the severity of COVID-19 in children.

Many machine learning models have been developed to predict the severity of COVID-19. Most of these works focus on adult patients (Bennett et al., 2021; Pitts et al., 2022; Schmidt et al., 2021). Some works conduct statistical analyses to identify potential risk factors for pediatric patients on the large multi-site Electronic Health Record (EHR) dataset (Martin et al., 2022; Nachega et al., 2022). Other works apply statistical models, such as the multi-variable Bayesian model, to single-site pediatric patient cohorts to predict severity with limited features (Domínguez-Rodríguez et al., 2021; Oliveira et al., 2022). However, the complex relationships within such heterogeneous data limit the feasibility of statistical approaches. It may be possible to develop more accurate models with specific machine learning techniques designed for this purpose, but this presents two major technical challenges to be solved:

1. How can we fuse clinical domain knowledge and data-driven discovery? Most existing EHR-based machine learning models use features primarily selected from lab tests and diagnosis codes. If data from multiple sites are used, the data need to be homogenized so that labels are consistent across all data sources (e.g., if two hospitals have different units for the C-reactive protein test, the data need to be converted). Model complexity grows immensely as the number of input features increases, so limiting the number of features can lead to a simpler, faster model that is easier to deploy. For large national EHR datasets, the number of medical features is much greater. In the case of the publicly available National Cohort Collaborative (N3C) COVID-19 dataset (Haendel et al., 2021), more than 100,000 heterogeneous features have been collected from many care sites to create a diverse dataset of outpatient and inpatient care datasets. Existing machine learning models do not perform as well with N3C data because the inputs are too sparse and noisy. There are two primary methods of addressing this issue: knowledge-driven selection and data-driven selection. Knowledge-driven selection is most useful if the number of features is small and the relative importance of each feature is already known. Some existing works address the complexity of large, feature-rich clinical datasets through a manual selection of clinical features using domain knowledge (Bennett et al., 2021; Elliott et al., 2021). This approach reflects clinician insight, but if the model is not allowed to learn independently, it becomes constrained by that knowledge. If a clinical element is not included (or is simply unknown), it cannot be discovered. To solve this problem, data-driven models can be used to identify which features are most relevant to the task at hand based on the model’s performance using those features. Identifying the unknown patterns can therefore be useful for clinical teams in three ways: 1) it may identify features that were omitted from the knowledge-driven model; 2) it may uncover errors in the data; and 3) it may discover strong correlations that were previously unknown and may merit further exploration. Therefore, fusing domain knowledge and data-driven models is an exciting opportunity to enhance model performance and even discover new clinical insights through data.
2. How can we model the relationships between aspects of clinical knowledge? EHR data consist of multiple data types, including medications, measurements, procedures, diagnoses, etc. The relationships between these data elements range from simple paired correlation to highly complex multi-variable associations, neither of which is captured by traditional knowledge-based or data-driven modeling. Fusing these complex relationships can guide the model to extract complex patterns in data and lead to better prediction performance. Utilizing the existing clinical knowledge also increases the trustability of the model because the decision process is much more traceable than the purely “black box” performance of other deep learning methods.

To address the above two challenges, we suggest a hybridized approach of constructing the model from a framework of clinical domain expertise in the form of a knowledge graph, and then allowing the model to improve through data-driven exploration. Our proposed model, MedML, fuses medical knowledge graphs and data-driven feature extraction to better predict pediatric COVID-19 hospitalization and severity. Specifically, MedML translates expert knowledge into clinical knowledge graphs, which map granular medical features to clinical cohorts and connect these cohorts with explicit graphical relationships. The use of knowledge graphs for generating patient EHR embeddings ensures that domain knowledge can directly guide the learning process and in turn improve clinical understanding. Integrating known expert knowledge and clinical decision-making processes into deep learning models also allows us to filter out irrelevant or noisy inputs, resulting in a leaner, more efficient model that can be orders of magnitude faster than high-feature models, while still achieving comparable or even superior prediction accuracy. MedML also enriches the feature sets using data-driven extraction methods based on propensity scores, which leads to more accurate predictions.

**Figure 1:**
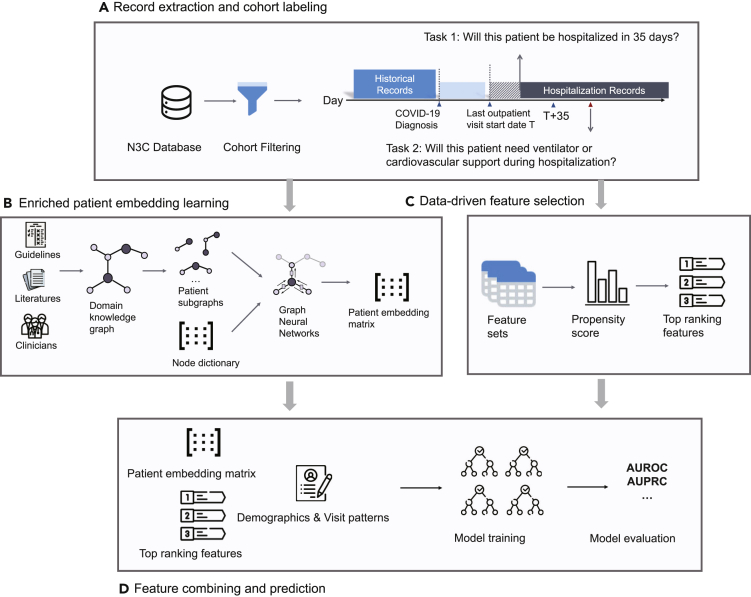
The proposed MedML pipeline (A) Record extract and cohort labeling. We extract patient records from the N3C database. We then filter and label patient cohorts using our task definitions. (B) Learn enriched patient embeddings by applying graph attention networks (GAT) on domain knowledge graphs. Each patient is represented as a subgraph of the knowledge graph. We use GATs to learn the hierarchical relationships between medical concepts and generate the final enriched patient embedding matrix. (C) Data-driven feature selection. Simultaneously, we use the propensity score to filter the most predictive medical features as the augmented feature set. (D) Feature combining and prediction. We combine the patient graph embedding matrix, data-driven features with the highest propensity scores, patient demographics, and visit patterns to train a gradient boost model. Both tasks follow the same pipeline, albeit with different patient cohorts.

Our overall pipeline can be divided into four modules: (1) data extraction and cohort labeling, (2) enriched patient embedding using graph attention networks on the constructed medical knowledge graphs, (3) data-driven feature selection, and (4) feature combining and prediction. The overall pipeline illustration is shown in Figure 1.

## Results

### Dataset and task description

We use the COVID-19 pediatric cohort from the National COVID Cohort Collaborative (N3C) data enclave (Haendel et al., 2021). The enclave includes demographic and clinical characteristics of patients who have been tested for or diagnosed with COVID-19. It also provides information about the strategies and outcomes of treatments for those suspected or confirmed to have the virus. As of Feb 2022, this enclave has 4 million COVID cases, over 1200 million clinical observations, and 14 billion record rows.

We develop our MedML model for pediatric COVID-19 prediction tasks by fusing medical knowledge and data-driven machine learning approaches. We formulate the model inputs and prediction tasks as follows:

#### Definition 1 (patient records)

Each patient record is represented as a vector p. Each feature dimension in p denotes a patient EHR indicator, including disease and procedure occurrence, lab test values, and drug usage, as well as static features including patient demographics.

#### Definition 2 (medical knowledge graph)

A medical knowledge graph G(V,E) is constructed by domain experts based on clinical practice from physicians and relevant published research. G is a directed hierarchical graph representing direct and indirect risk factors with multiple levels. We use v to denote the node embedding of a medical feature in the node set V. Edge e between node i and node j indicates that the two features are related. We will introduce the detailed construction process in the method section.

We aim to predict hospitalization and severity risk for pediatric patients with COVID-19. First, we identify the patients at the risk of hospitalization from all COVID-19-positive pediatric patients. Then, we identify the patients at the risk of severe outcomes from all hospitalized patients. To perform an early-stage prediction on high-risk patients, we only use the data available at the outpatient visit start date or hospitalization date for two pediatric prediction tasks. The concrete problem formulations are shown below:

#### Task 1 (pediatric COVID-19 patient hospitalization risk prediction)

Given a COVID-19-positive pediatric patient with EHR data p on the first day of outpatient visit after the COVID-19 diagnosis, our target is to predict the hospitalization risk $y_{h}$ in the coming 35 days. We formulate this task as a binary classification task as $\;y_{h}\in \left\{0,1\right\}$.

#### Task 2 (pediatric COVID-19 patient severity prediction after hospitalization)

If a pediatric patient with COVID-19 is hospitalized, given EHR data p on the first day of hospitalization, we further predict the risk of getting severe outcomes (reflected by needing ventilation, cardiovascular interventions, ECMO, or death) $y_{s}$ during that encounter. We identify ventilated patients based on ventilation procedure codes in their encounter record and cardiovascular support based on the presence of the injectable forms of any of the following medications: dobutamine, dopamine, epinephrine, levosimendan, milrinone, norepinephrine, phenylephrine, and vasopressin. The task is formulated as a binary classification such that $y_{s}\in \left\{0,1\right\}$, and our working clinical definition for severe outcomes is consistent with existing works (Bennett et al., 2021). This task is also formulated as a binary classification task as $y_{s}\in \left\{0,1\right\}$.

The notations used in this study are listed in the Table S1.

### Cohort construction and evaluation metrics

**Figure 2:**
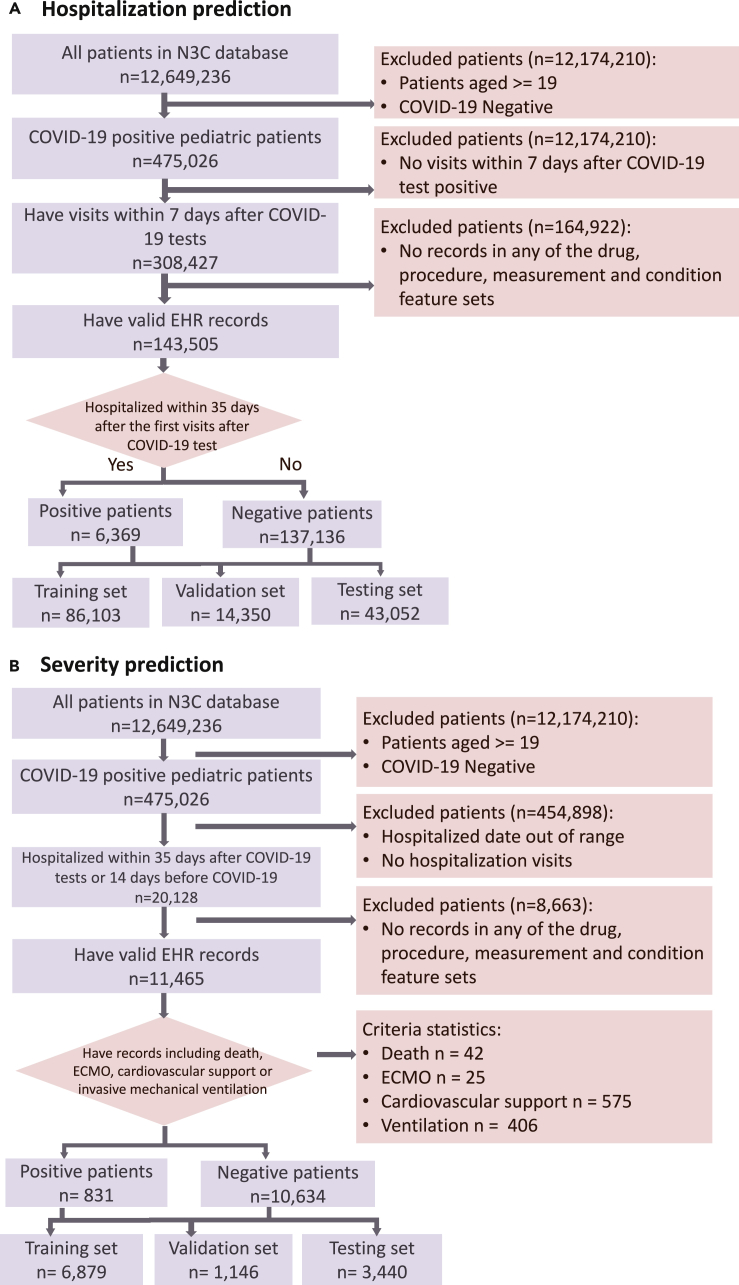
Patient cohort construction flow chart (A) Flow chart of the hospitalization prediction patient cohort. (B) Flow chart of the severity prediction patient cohort.

We define COVID-19-positive patients as patients who have received the positive COVID-19 PCR test. We conduct two prediction tasks—hospitalization prediction and severity prediction—and both are binary prediction tasks. In this study, we include patients from 2020/01/01 to 2022/02/01 to build the patient cohorts. The visit information is extracted from the visit record table. The patient cohort construction process of the two tasks is inspired by the pediatric COVID-19 data challenge (The BARDA Community Challenge, 2022) and the pipelines are shown in Figure 2. In the severity prediction task, we include the patients hospitalized not only within 35 days after the COVID-19 diagnosis but 14 days before the diagnosis following the challenge cohort definitions. This is to ensure all target patients are included, since some patients are infected during the hospitalization or do not get diagnosed before hospitalization. The basic statistics of the extracted cohorts are shown in Tables 1 and 2.

**Table 1:** Patient characteristics for the hospitalization prediction task

	Total	Negative (%)	Positive (%)
Number of patients	143,505	137,136 (95.6%)	6,369 (4.4%)

**Age**			

0–1	3,591 (2.5%)	3,274 (91.2%)	317 (8.8%)
1–3	12,043 (8.4%)	11,195 (93.0%)	848 (7.0%)
3–6	15,569 (10.8%)	14,840 (95.3%)	729 (4.7%)
6–12	39,217 (27.3%)	38,033 (97.0%)	1,184 (3.0%)
12–18	73,085 (50.9%)	69,794 (95.5%)	3,291 (4.5%)
Mean (Median)	10.8 (12.0)	10.8 (12.0)	10.3 (12.0)

**Gender**			

Male	71,413 (49.8%)	68,380 (95.8%)	3,033 (4.2%)
Female	72,069 (50.2%)	68,733 (95.4%)	3,336 (4.6%)
Others/Unknown	23 (0.01%)	23 (100.0%)	0 (0.00%)

**Race**			

White	99,832 (69.6%)	95,574 (95.7%)	4,258 (4.3%)
Black	17,475 (12.2%)	16,420 (94.0%)	1,055 (6.0%)
Asian	2665 (1.9%)	2,582 (96.9%)	83 (3.1%)
Asian/Pacific	846 (0.6%)	817 (96.6%)	29 (3.4%)
Others/Unknown	22,687 (15.8%)	21,743 (95.8%)	944 (4.2%)

**Ethnicity**			

Not Hispanic or Latino	103,905 (72.4%)	99,178 (95.5%)	4,727 (4.5%)
Hispanic or Latino	27,717 (19.3%)	26,437 (95.4%)	1,280 (4.6%)
Others/Unknown	11,883 (8.3%)	11,521 (97.0%)	362 (3.0%)

**Visit statistics - Mean (Median)**			

# of visits	30.1 (17)	28.9 (16)	43.8 (24.3)
Avg. days between visits	75.2 (44.1)	76.7 (45.3)	55.1 (27)

**Table 2:** Patient characteristics for the severity prediction task

	Total	Negative (%)	Positive (%)
Number of patients	11,465	10,634 (92.8%)	831 (7.2%)

**Age**			

0–1	950 (8.3%)	917 (96.5%)	33 (3.5%)
1–3	1,758 (15.3%)	1,654 (94.1%)	104 (5.9%)
3–6	1,265 (11.0%)	1,162 (91.9%)	103 (8.1%)
6–12	2,102 (18.3%)	1,917 (91.2%)	185 (8.8%)
12–18	5,390 (47.0%)	4,984 (92.5%)	406 (7.5%)
Mean (Median)	9.6 (11.0)	10.1 (11.0)	9.5 (11.0)

**Gender**			

Male	5,551 (48.4%)	5,103 (91.9%)	448 (8.1%)
Female	5,914 (51.6%)	5,531 (93.5%)	383 (6.5%)

**Race**			

White	5,597 (48.8%)	5,222 (93.3%)	375 (6.7%)
Black	3,060 (26.7%)	2,808 (91.8%)	252 (8.2%)
Asian	204 (1.8%)	183 (89.7%)	21 (10.3%)
Asian/Pacific	52 (0.5%)	52 (100%)	0 (0%)
Others/Unknown	2,552 (22.3%)	2,369 (92.8%)	183 (7.2%)

**Ethnicity**			

Not Hispanic or Latino	7,857 (68.5%)	7,305 (93.0%)	552 (7.0%)
Hispanic or Latino	2,929 (25.5%)	2,716 (92.7%)	213 (7.3%)
Others/Unknown	679 (5.9%)	613 (90.3%)	66 (9.7%)

**Visit statistics - Mean (Median)**			

# of visits	50.9 (20)	48.4 (19)	83.2 (26)
Avg. days between visits	57.7 (24.6)	57.9 (25.3)	55.1 (16.1)

**Figure 3:**
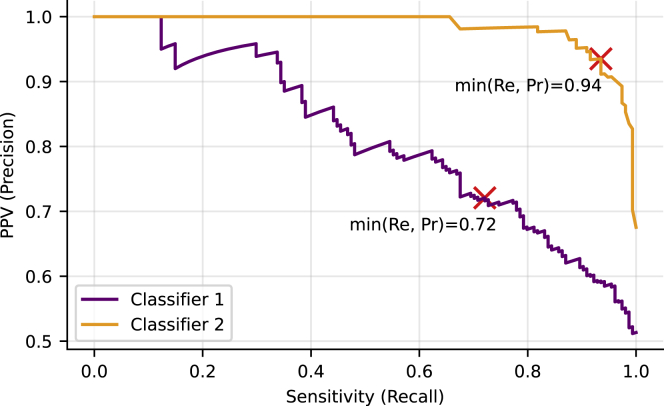
An example of Min(Re,Pr)

For experimental setup, two cohorts are split into training, validation, and testing sets by 6:1:3. We use the training set to fit the models and use the validation set to determine the hyper-parameters. Finally, we evaluate the models on the testing set and report the prediction performance. The evaluation metrics are AUROC (area under the receiver operating characteristic curve), AUPRC (area under the precision-recall curve), and the maximum of the minimum between precision and sensitivity under the same threshold, known as Min(Re,Pr), following existing works (Ma et al., 2020, 2021). An example of Min(Re,Pr) is shown in Figure 3, which evaluates whether the model can achieve the balance between precision and recall. All three metrics are higher the better. Calibration of models was examined to assess how similar the model’s probability predictions are to the observed probabilities for severity and hospitalized COVID-19 cases (Huang et al., 2020). We also evaluate the final model’s calibration performance using calibration curves.

### Baselines and experiment settings

We first compare the proposed MedML with its different variant versions to validate the predictive performance of different feature sets:

1. **MedML (Propensity)**: We remove the knowledge graph embedding g from the overall feature set.
2. **MedML (Domain)**: We remove the data-driven feature m and replace graph embedding g with the simple concatenation of node features.
3. **MedML (KG)**: We remove the data-driven features m from all features.

**Figure 4:**
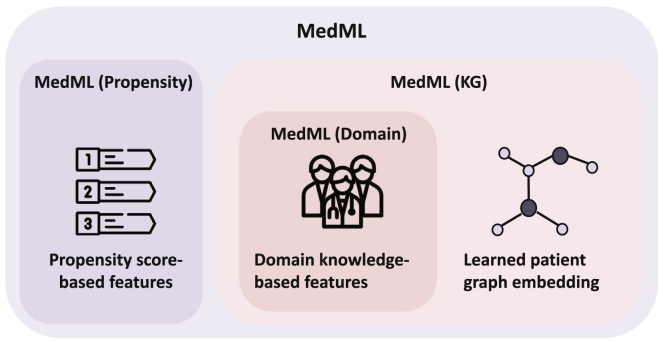
Features used in MedML and its variants All models will use patient demographics and visit sequence features.

The illustration of different feature sets used in these variants is shown in Figure 4.

The hyperparameters of these models are decided to maximize the highest score (the average of three metrics, the same below) on the validation set. We set the node embedding dimension E to 128. The hidden dimension of the GAT model and the MLP dimension are set to 128. To evaluate the predictive performance of the data-driven features fairly, we also compare MedML with several well-known machine learning and deep learning baseline classifiers, which are widely applied in EHR predictive modeling including COVID-19-related prediction tasks. These models are trained with the data-driven feature set m, demographics, and visit features s.

#### XGBoost (XGB) and decision tree (DT)

XGBoost (Chen and Guestrin, 2016) is a popular and efficient gradient-boosted trees algorithm. We select hyperparameters that maximize the highest score (the average of three metrics) on the validation set. XGBoost and decision tree models have been applied in hospitalization and severity prediction tasks for adult patients with COVID-19 (Bennett et al., 2021; Yan et al., 2020).

#### Logistic regression (LR)

We use L2 regularization for this baseline and the regularization hyperparameter is selected by maximizing the highest score on the validation set, which is set 0.7. Since the logistic regression model may have difficulty converging on the highly imbalanced data, we set the class weight to 0.3 for the hospitalization task and 0.15 for the severity prediction task. The LR model has also been applied in COVID-19 risk prediction tasks (Elliott et al., 2021).

#### Multi-layer perceptron (MLP)

Multi-layer perceptron is a general deep learning classifier. We use a two-layer perceptron to achieve classification. The layer dimension is set to maximize the highest score on the validation set. The hidden dimension of the MLP model is set to 128.

The final dimension of g and s is 128 and 90, respectively. The dimension of m depends on how many features we selected from the propensity-based feature list (i.e., K). We have a detailed analysis for the selection of K in the result section.

For LR, XGBoost, and DT models, we use the scikit-learn package (Pedregosa et al., 2011) (version 0.23.2) and XGBoost (Chen and Guestrin, 2016) (version 1.0.2) to construct and train the models. For MLP and MedML models, we use the PyTorch (Paszke et al., 2019) (version 1.7.1) to implement the models. We use the mini-batch gradient descent to train the models and the batch size is set to 128. We use Adam optimizer with learning rate 0.001 to train the models. The model training process was stopped if the score on the validation set did not improve over 30 consecutive iterations. All the experiments are done on the N3C Data Enclave with Code Workbook. The training process is accelerated using a Tesla T4 GPU. We save the model with the highest score on the validation set and report the prediction performances on the testing set.

### Model performance analysis

We design the experiments to discuss the following research questions and topics:

1. How does the proposed MedML perform on two pediatric COVID-19 prediction tasks?
2. How does the number of features affect the performance of MedML and baselines?
3. How does MedML perform in different time periods and geographic locations?
4. Analysis for data-driven features, demographic features, and knowledge graph features.

#### Performance on pediatric COVID-19 prediction tasks

The prediction performance of MedML and baseline models on the two tasks is shown in Tables 3 and 4. For the number of input features, MedML, knowledge-based baselines (MedML (Domain), MedML (KG)), and other propensity score-based baselines (XGB, DT, MLP, LR, and MedML (Propensity)) use different feature sets. For example, the number of input features for knowledge graph-based models is fixed (65 features), while the number for propensity score-based models depends on the hyperparameter K. To achieve a fair comparison, we set K to 80 for propensity score-based models and K to 15 for the MedML model, so that these models will be accessing the same number of features (except for two variants, MedML (Domain) and MedML (KG), which only uses 65 knowledge-based features).

**Table 3:** Performance comparison for pediatric COVID-19 hospitalization prediction

Model	AUROC	AUPRC	Min(Re, Pr)
XGB	0.7321 (0.002)	0.1302 (0.003)	0.1660 (0.003)
DT	0.6232 (0.002)	0.1198 (0.002)	0.0915 (0.002)
MLP	0.7228 (0.003)	0.1439 (0.003)	0.1770 (0.003)
LR	0.6501 (0.002)	0.1222 (0.001)	0.0928 (0.001)
MedML (Propensity)	0.7446 (0.005)	0.1420 (0.003)	0.1916 (0.003)
MedML (Domain)	0.7204 (0.004)	0.1370 (0.003)	0.1832 (0.002)
MedML (KG)	0.7294 (0.002)	0.1432 (0.002)	0.1879 (0.004)
MedML	**0.7544∗** (0.003)	**0.1501∗** (0.002)	**0.1982∗** (0.003)

The asterisk ∗ denotes the performance differences between MedML and the best baseline models are significant based on the t-test results (p < 0.001).

**Table 4:** Performance comparison for pediatric COVID-19 severity prediction

Model	AUROC	AUPRC	Min(Re, Pr)
XGB	0.6477 (0.002)	0.1605 (0.005)	0.2049 (0.004)
DT	0.5439 (0.003)	0.1599 (0.004)	0.1326 (0.002)
MLP	0.6395 (0.002)	0.1928 (0.003)	0.1993 (0.003)
LR	0.5448 (0.002)	0.1307 (0.004)	0.1621 (0.004)
MedML (Propensity)	0.6519 (0.004)	0.1615 (0.005)	0.2048 (0.004)
MedML (Domain)	0.6822 (0.002)	0.1810 (0.004)	0.2369 (0.003)
MedML (KG)	0.6915 (0.003)	0.2162 (0.003)	0.2693 (0.003)
MedML	**0.6926∗** (0.002)	**0.2202∗** (0.004)	**0.2701∗** (0.004)

The asterisk ∗ denotes the performance differences between MedML and the best baseline models are significant based on the t-test results (p < 0.001).

MedML achieves the best performance on both tasks in terms of all three metrics. For the pediatric COVID-19 hospitalization prediction task, MedML achieves 3% higher AUROC, 4% higher AUPRC, and 12% higher min(Re, Pr) compared to the best machine learning models, XGB and MLP, which utilize propensity score-based features. For the pediatric COVID-19 severity prediction task, the performance gap is much larger. Compared with the best baselines, MedML achieves 7% higher AUROC, 14% higher AUPRC, and 32% higher min(Re, Pr). We conduct the Student’s *t* test to evaluate the significance of the performance differences. The results show that the performance differences between MedML and the best baseline models are significant (p < 0.001).

MedML also outperforms its variants. We note that on the severity prediction task, using the same GBDT model, models built on knowledge graph features (MedML (Domain) and MedML (KG)) outperform the model with propensity score-based features (MedML (Propensity)) in all three metrics. MedML (KG) consistently outperforms MedML (Domain) on both tasks, which suggests that utilizing the structured inter-feature relationships in the knowledge graph (MedML (KG)) further improves the prediction performance.

For baseline models, we find that deep learning-based and ensemble-based models (MLP and XGB) have better performance than LR and DT on both tasks. This may be because the high feature dimension and data imbalance make it difficult for naive machine learning models to learn complex patterns. Generally, we find the hospitalization prediction task is a more challenging task for machine learning models in terms of AUPRC and min(Re, Pr) metrics, which are more informative metrics in this data imbalance setting (Powers, 2011). This is primarily because of the noise in the labels and data. The severity labels (ECMO, ventilator, death, and cardiovascular interventions) indicate a patient’s health status is at high risk and their status is much more distinctive. While the hospitalization criteria for patients are more diversified, that diversity makes it more difficult for the model to decide whether the patient should be hospitalized.

**Figure 5:**
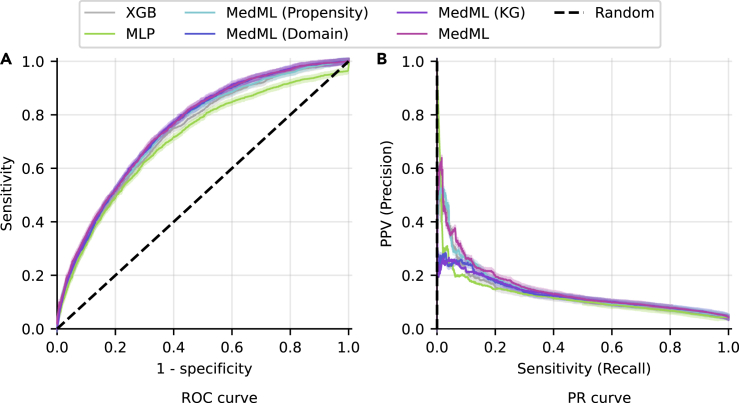
The ROC curve and PR curve for the hospitalization prediction task The ROC curve (A) and PR curve (B) for the hospitalization prediction task

**Figure 6:**
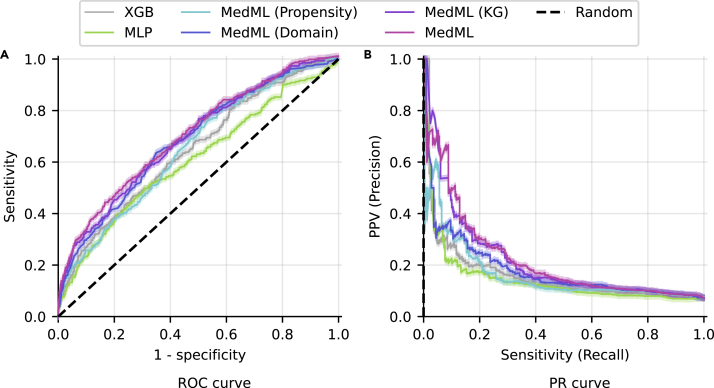
The ROC curve and PR curve for the severity prediction task The ROC curve (A) and PR curve (B) for the severity prediction task.

The ROC curve and PR curve of both tasks are shown in Figures 5 and 6. For simplicity, we only show the curves for XGB and MLP among all baseline models, which achieve better performance in both tasks.

#### Predictive performance under different K

**Figure 7:**
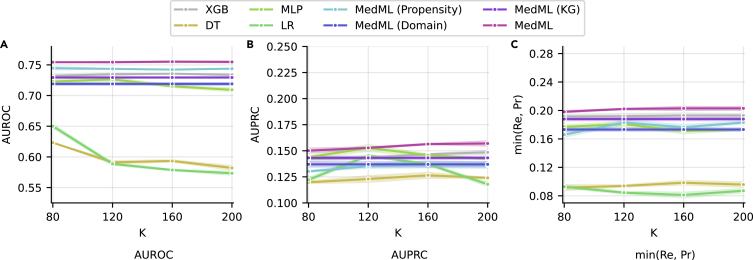
Hospitalization prediction performance under different K from 80 to 200 Hospitalization prediction performance under different K from 80 to 200 in AUROC (A), AUPRC (B) and min(Re, Pre) (C).

**Figure 8:**
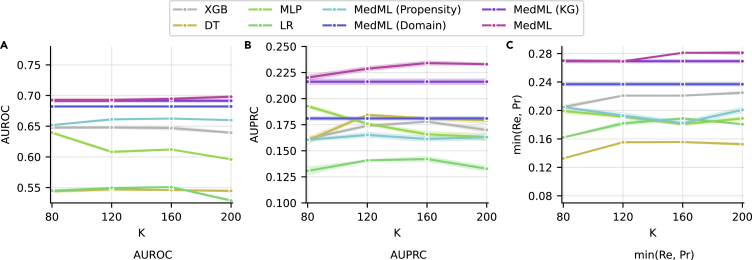
Severity prediction performance under different K from 80 to 200 Severity prediction performance under different K from 80 to 200 in AUROC (A), AUPRC (B) and min(Re, Pre) (C).

To evaluate the model performance with different numbers of features, we study the effect of different K from 80 to 200. We also keep the total number of features the same for different models for a fair comparison. The results are shown in Figures 7 and 8. For MedML (Domain) and MedML (KG), they only use knowledge graph features, so their performance will not be affected by K and their plots are therefore straight lines in the figures.

Note that MedML achieves the best performance on all metrics for all values of K. The performance gap between MedML and other propensity score-based models is much larger on the severity prediction task. As K increases, the newly included features will have relatively lower propensity scores, which means lower distinctiveness. It is interesting to find that including more features hinders the performance of some baseline models, such as LR and DT. The worse performance is caused by high feature dimensionality with the increasing number of features.

Also note that compared with models using 200 propensity score-based features, MedML (KG) can still achieve almost equivalent performance on the hospitalization prediction task and outperform all other models on the severity prediction task only using 65 knowledge graph features. This suggests that fusing domain knowledge into machine learning models and learning the inter-relationships between features can help reduce the feature set size while maintaining good predictive performance. This reduces computational load and improves the reliability of the model while still maintaining accurate predictions.

#### Prediction performance under temporal data split setting

**Figure 9:**
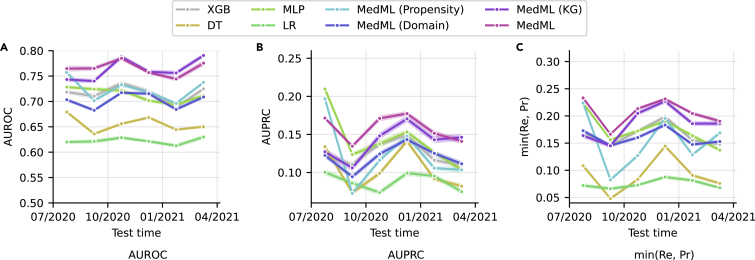
Hospitalization prediction performance under different time split Hospitalization prediction performance under different time split in AUROC (A), AUPRC (B) and min(Re, Pre) (C).

**Figure 10:**
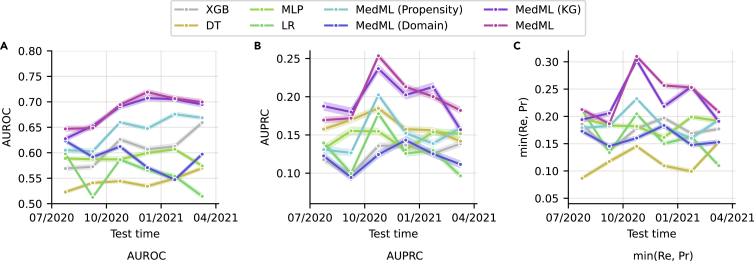
Severity prediction performance under different time split Severity prediction performance under different time split in AUROC (A), AUPRC (B) and min(Re, Pre) (C).

We design experiments to evaluate how model performance evolves over time. We split the time from 01/2020 to 01/2022 into 8 3-month periods. Each patient will be assigned to the corresponding period based on the COVID-19 diagnosis date. We use patient data from the consecutive two periods to train the model, then test the model on the next period. For example, we train the model using data from 01/01/2020 to 06/30/2020 and subsequently test the model on data from 07/01/2020 to 09/30/2020. This process is conducted in a rolling manner, which means we will test the model on each 3-month period from 07/01/2020 to 12/31/2022. The results for both tasks are shown in Figures 9 and 10.

**Figure 11:**
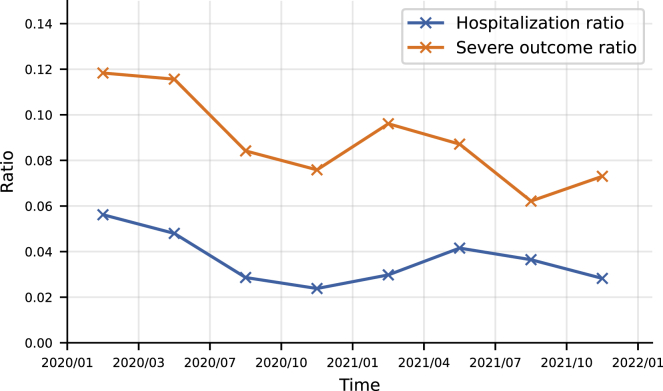
Positive patient ratio across two tasks

We find that for the hospitalization prediction task, MedML and MedML (KG) outperform all baseline models in all periods except for the earliest stage of the pandemic (2020/07–2020/12). For the severity prediction task, MedML and MedML (KG) generally outperform all other baselines in all periods. MedML generally performs better than MedML (KG) on most metrics. The relatively lower early-stage performance reflects the greater effectiveness of propensity score-based features than domain knowledge extracted by clinicians in the early stage of the pandemic. However, with the virus evolving and the pandemic progressing, the domain knowledge-based features are consistently predictive throughout all subsequent pandemic stages. At the later stage of the pandemic (2021/07–2022/12), the performance of MedML and MedML (KG) drops slightly, which seems to correlate with the lower positivity rate that results in a more unbalanced training set. The positive patient ratios are shown in Figure 11.

#### Prediction performance under geographical data split setting

**Figure 12:**
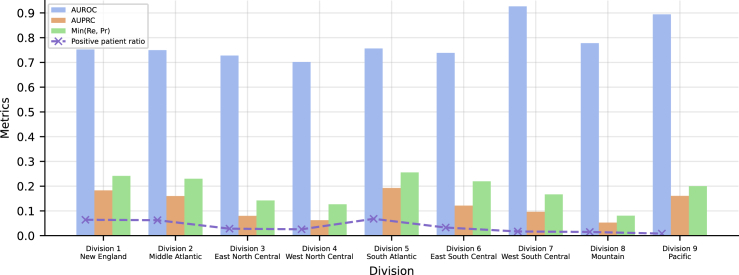
Hospitalization prediction performance visualization in different divisions

**Figure 13:**
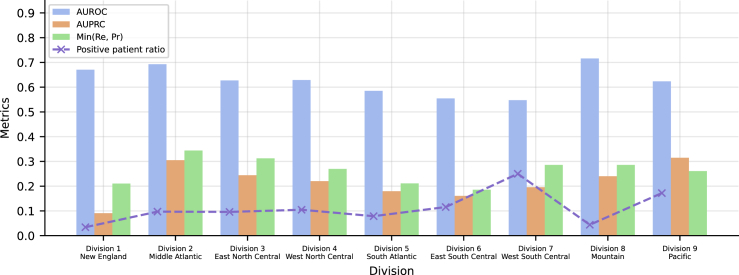
Severity prediction performance visualization in different divisions

We also design experiments to evaluate the geographical generalizability of MedML. We divide patients into different geographical regions based on their location information. We use 9 divisions based on US bureau divisions (The United States Census Bureau, 2022). We do not aggregate patients at the state level because the number of patients is too small to train the model. We use the leave-one-out strategy to evaluate our model, which means training MedML on 8 divisions and testing the model on the last division. We visualize the prediction performance of MedML and the positive patient ratio in different regions in Figures 12 and 13.

MedML performs better in division 9 Pacific, division 5 South Atlantic, and division 2 Middle Atlantic for both tasks compared to other divisions. For the severity prediction task, MedML performs worse in Division 1 New England. This may be because the positive ratio of this division is significantly lower than other divisions, and the imbalanced data hinder the model.

#### Feature importance analysis

**Figure 14:**
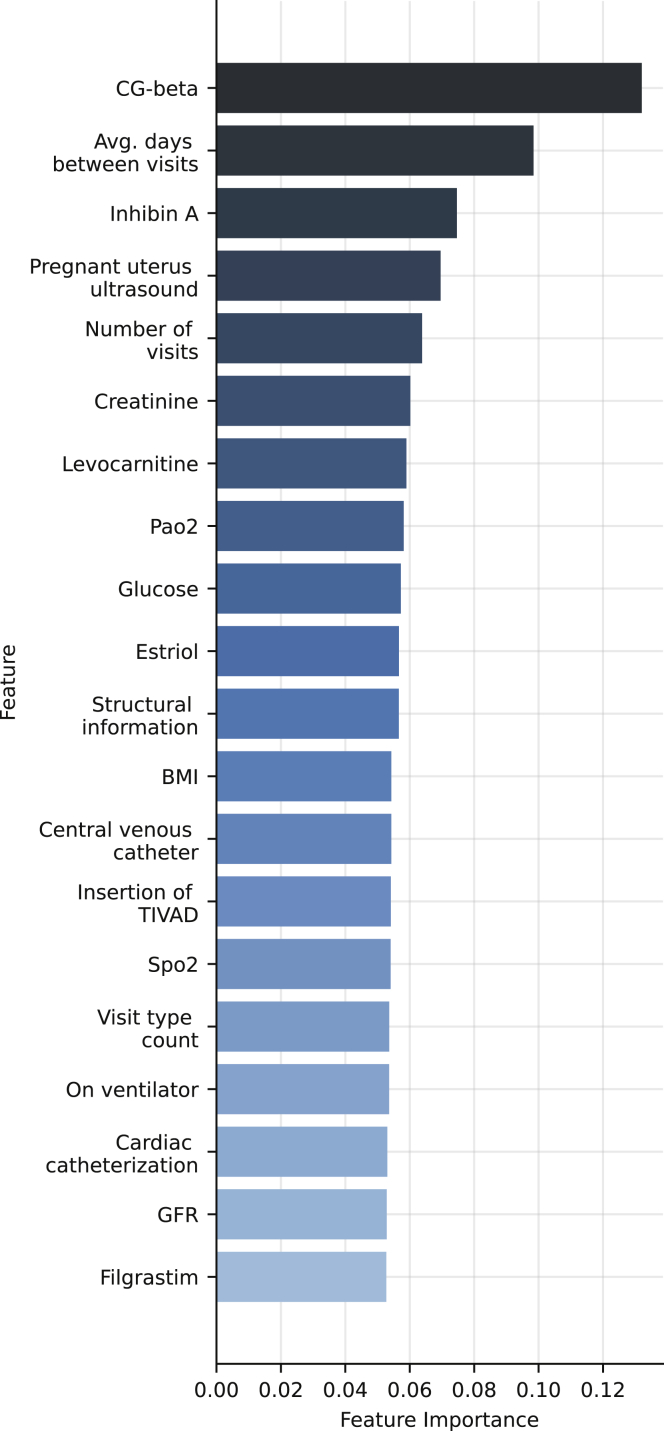
Feature importance in MedML for the hospitalization prediction task

**Figure 15:**
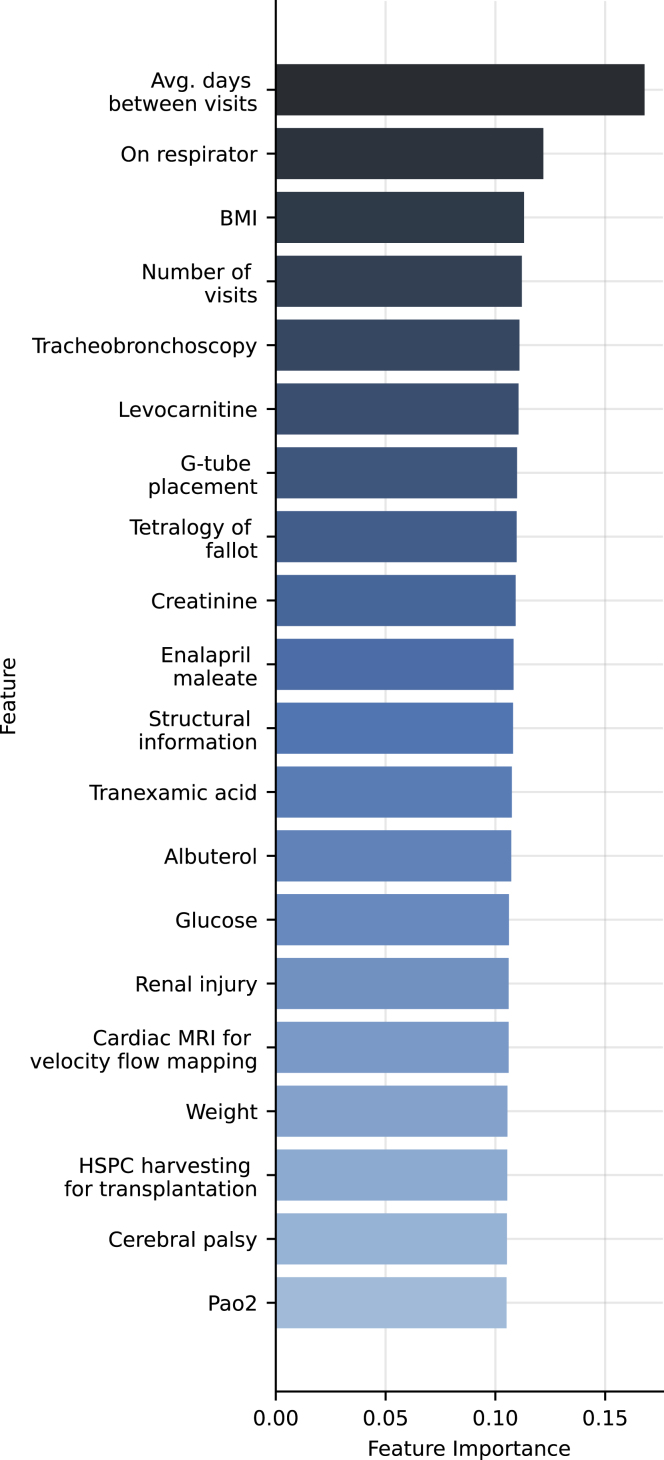
Feature importance in MedML for the severity prediction task

We list the top 20 features with the highest importance in MedML for both tasks in Figures 14 and 15. For a specific feature, its importance in MedML is calculated by measuring its average importance in different trees. Concretely, the feature importance $J_{i}$ of feature i is calculated as:

$$J_{i}=\frac{1}{M}\overset{M}{\underset{m}{\sum }}J_{i}\left(T_{m}\right)$$

$$J_{i}\left(T_{m}\right)=\overset{L-1}{\underset{t=1}{\sum }}I_{t}^{2}1\left(v_{t}=i\right)$$

where M denotes the number of trees, $J_{i}\left(T_{m}\right)$ denotes the feature importance in the m-th tree, L denotes the number of leaf nodes, $v_{t}$ is the feature related to node t, and $I_{t}^{2}$ is the reduced squared loss after further split.

In the figures, variables are ranked in descending order, with the darker color indicating a higher ability to predict each outcome. When predicting hospitalization risk, CG-beta (also known as beta-hCG) is the strongest predictor, followed by the average days between visits and Inhibin A. Similarly, when predicting severity, the average days between visits, ventilated status, and BMI were the strongest predictors. It is important to note that the factors identified in this study are correlated with but not necessarily causative of COVID-19 hospitalization and severity among the pediatric population.

**Figure 16:**
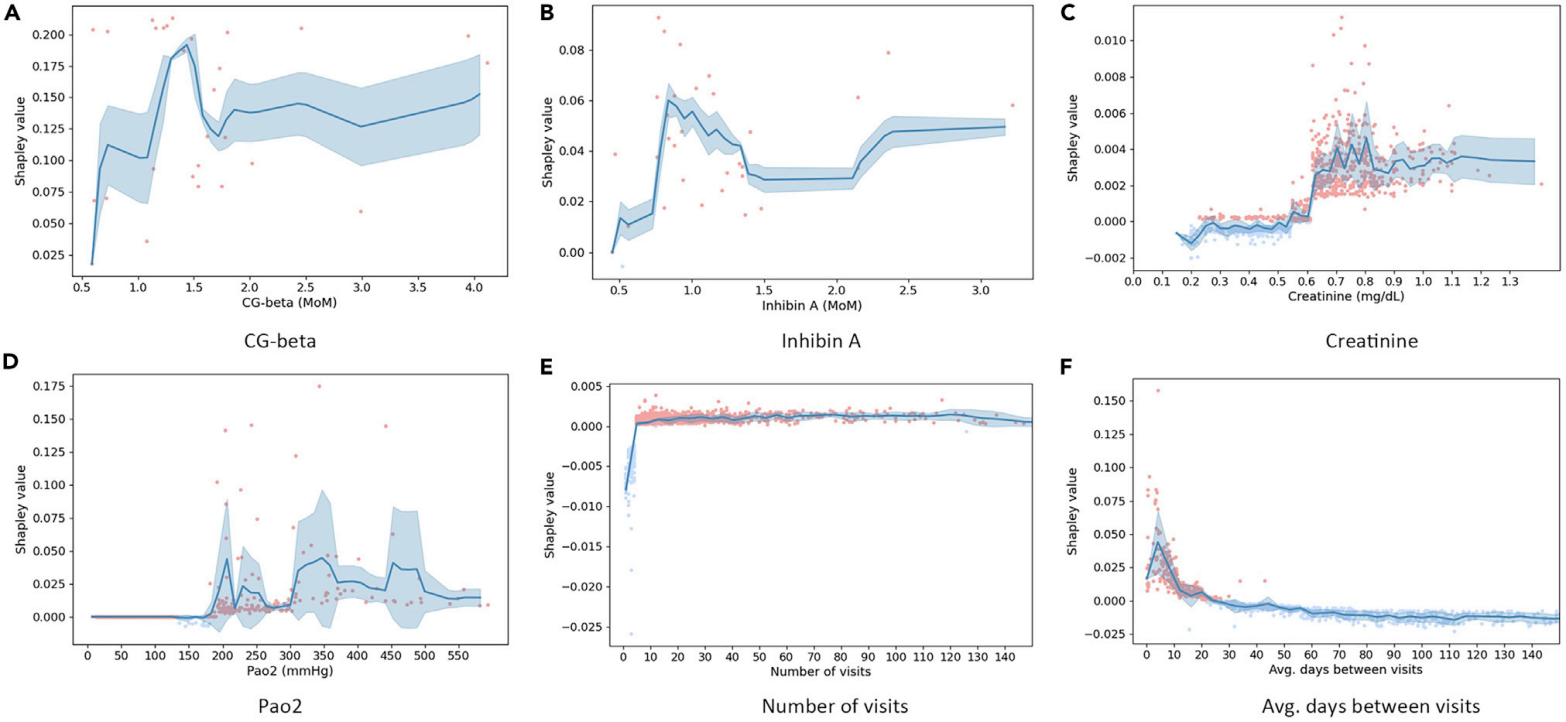
Shapley values of measurement features with top 4 highest importance and visit sequence features for the hospitalization prediction task

**Figure 17:**
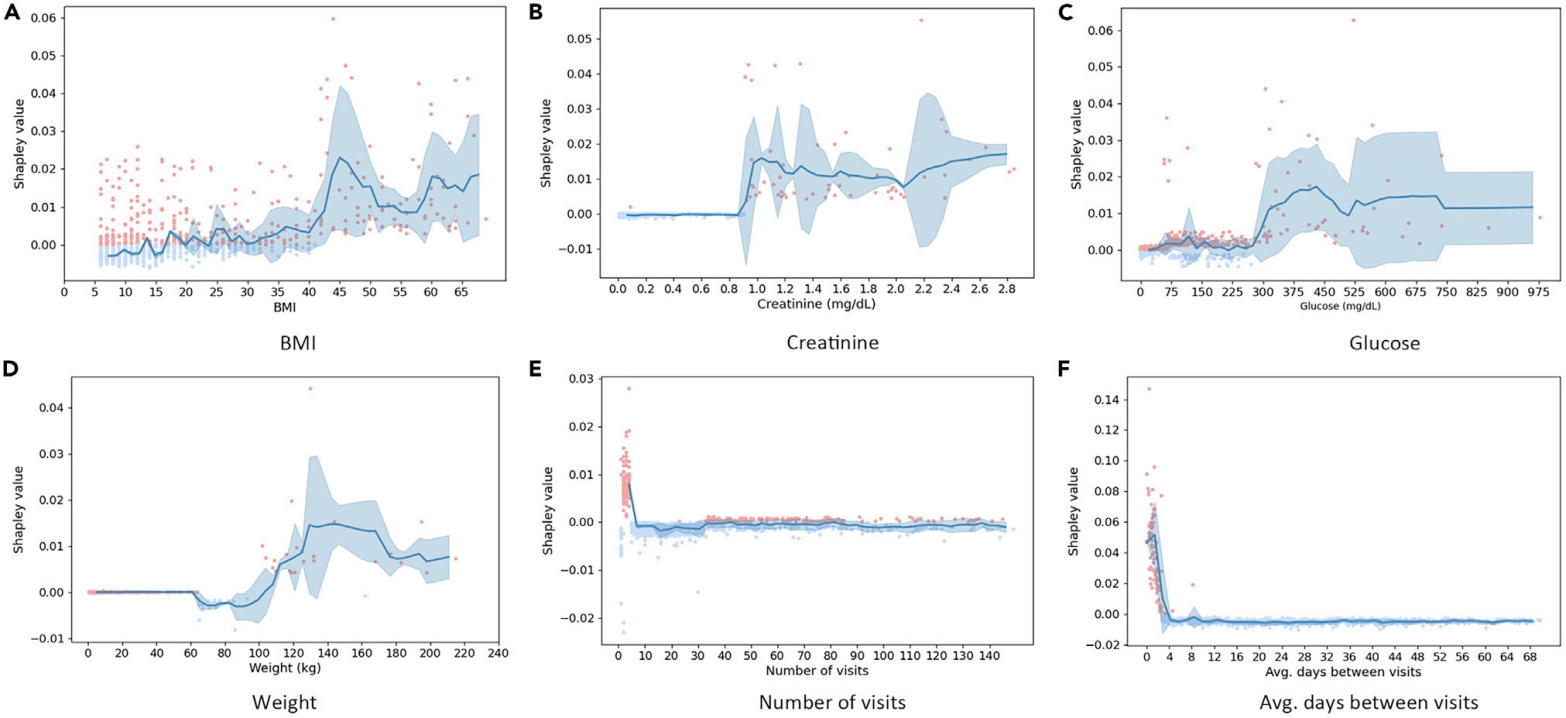
Shapley values of measurement features with top 4 highest importance and visit sequence features for the severity prediction task

The feature importance plots show that most of the predictive features in the two tasks are different. However, these tasks also share a few important features, e.g., levocarnitine, glucose, BMI, and PaO2. We find that the visit sequence-related features (i.e., average days between visits and number of visits) are highly important in both tasks. To further understand how feature values affect MedML’s output, we analyze the Shapley values of the measurement and visit pattern features. Shapley value is a common measurement of individual feature importance widely used in interpretability analysis for machine learning models (Hyland et al., 2020; Winter, 2002). A positive Shapley values means the feature is positively correlated with the target of interest, and higher values mean higher importance, while negative Shapley values correspond to negative correlations. We plot the Shapley value of measurement features with the top 4 highest importance, as well as average days between visits and number of visits. The results are shown in Figures 16 and 17.

In the figures, the x axis is the value of the figure, and the y axis is the Shapley value. Blue dots are COVID-19-negative patients and red dots are positive patients. For clarity, we only show 1000 random patients in the figure for some features. The blue line and shade are the means and standard deviations of the interpolated regression line. For the hospitalization prediction task, all four measurement features are positively correlated with the risk. For CG-beta and Inhibin A, the existence of these features positively contributes to the risk because all Shapley values are larger than 0. For creatinine and PaO2, there is a threshold between low-risk patients and high-risk patients, around 0.6 mg/dL and 200 mmHg, respectively. (Note: because PaO2 does not naturally occur at such high values, this is likely acting as a surrogate marker for patients on supplemental oxygen.) For the severity prediction task, BMI, creatinine, and glucose are positively correlated with the risk. The thresholds between low-risk patients and high-risk patients are 1.0 mg/dL for creatinine and 300 mg/dL for glucose. For weight, the model finds that patient’s weight between 60 and 100 kg have lower risk than the average. The risk increases as the weight increases beyond 100 kg.

For visit patterns, the model finds that for both tasks, patients with more frequent visits generally have higher hospitalization risk and have more severe outcomes. This is consistent with the statistical results in Tables 1 and 2. The statistics show that hospitalized and severe patients have more frequent visits (average 55.1 days) than low-risk patients (57.9–76.7 days). However, the total number of visit records have different effects on the two prediction targets. The model finds that patients with fewer recorded visits have a lower hospitalization risk, which is consistent with the statistics that demonstrate outpatients have, on average, less records (28.9) than hospitalized patients (43.8). For the severity, the plot shows that patients with a total number of visits between 10 and 40 have generally lower risks (Shapley value <0), but there are also some patients with higher Shapley values in this category as well. We conduct further analysis on the patients with visit records less than 5, and the results validate that these patients indeed have a higher severity ratio (9.8) than the average severity ratio (7.2). The reason might be that they are younger (8.0) than average (9.6), or they may have limited accessibility to medical resources.

#### Demographic patterns

**Figure 18:**
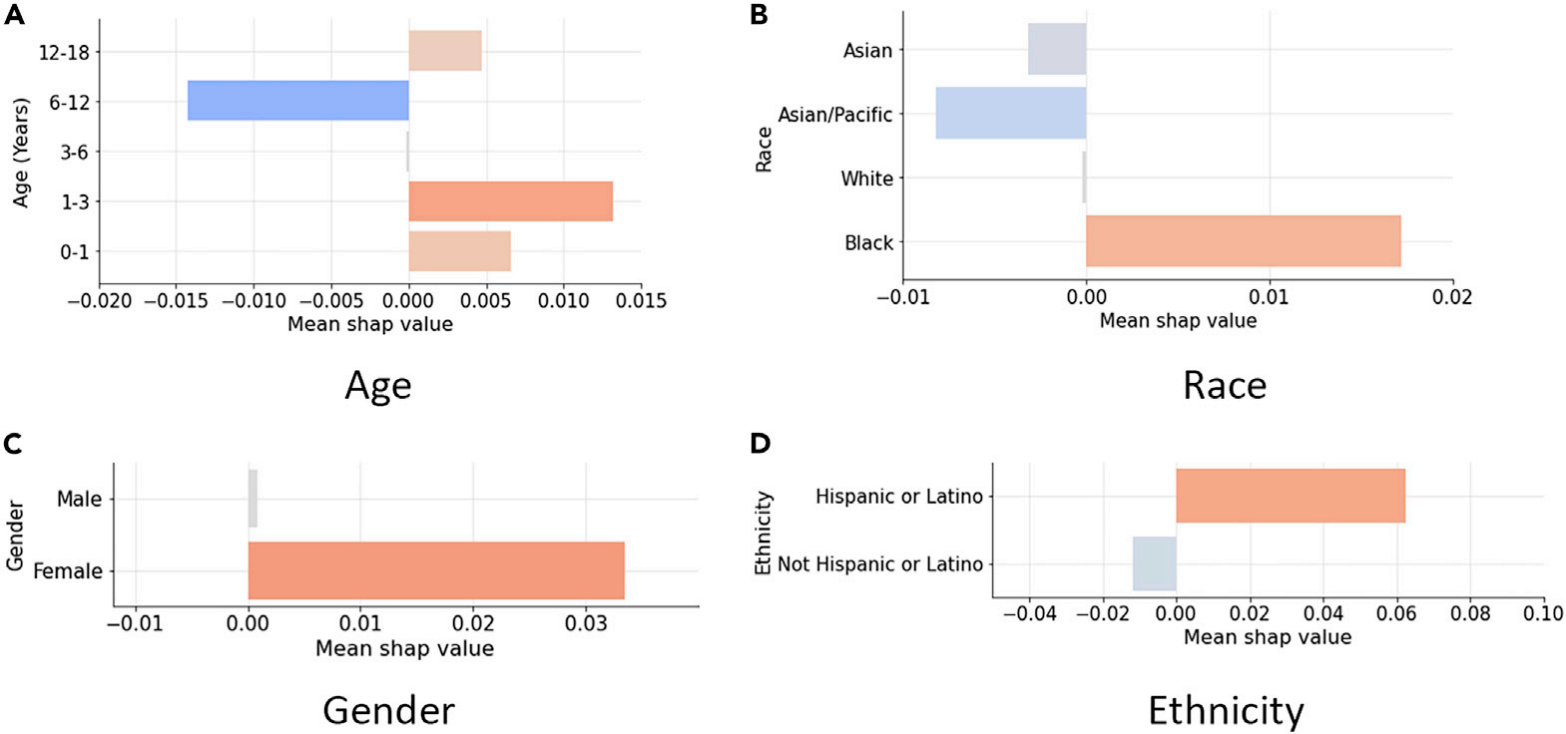
Shapley values of demographic features for the hospitalization prediction task

**Figure 19:**
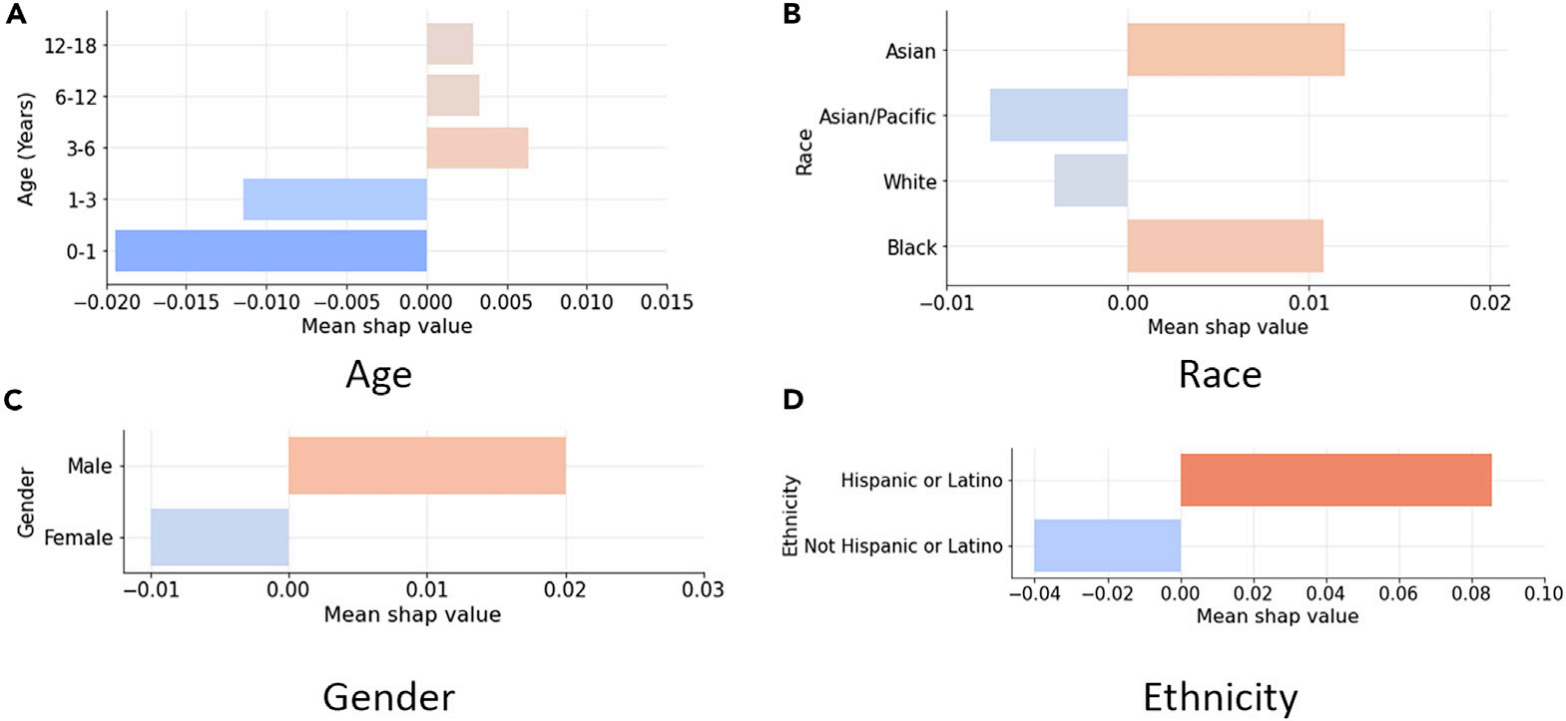
Shapley values of demographic features for the severity prediction task Shapley values of demographic features for the severity prediction task: A for Age, B for Race, C for Gender, and D for Ethnicity.

We also analyze MedML’s performance in different demographic cohorts. We plot the Shapley value of categorical demographic features including age, ethnicity, race, and gender for both tasks. The results are shown in Figures 18 and 19.

Different ages have different Shapley value distributions between the two tasks. For the hospitalization task, patients under 3 have the highest Shapley value (i.e., higher predicted hospitalization risk) and patients within 6–12 have the lowest Shapley value (i.e., lower predicted hospitalization risk). For the severity prediction task, the model finds that patients under 3 have the lowest severity while other patients have higher severity. These results are consistent with the statistic results in Tables 1 and 2. According to the statistic results, patients under 3 years of age have a higher hospitalization ratio (7%–9%) than the average hospitalization ratio (4.4%), but they have lower severity ratio (3%–6%) than the average severity ratio (7.2%). Patients between 6 and 12 indeed have lower hospitalization ratio (3.0%) and higher severity ratio (8.8%).

Gender also has different effects between the two tasks. The model finds that female patients are at higher risk for hospitalization but lower risk for severe outcomes than male patients. The hospitalization ratio of female patients is 4.6%, while the ratio for male patients is 4.2%. The severity ratio of female patients is 6.5%, while the ratio for male patients is 8.1%.

The model also finds that White/Caucasian patients generally have lower risks while Black/African American and Hispanic/Latinx patients have higher risks for both tasks. Asian patients, however, are at lower risk for hospitalization but higher risk for severe outcomes. The raw data statistics reveal the actual hospitalization ratio (White – 4.3%, Black – 6.0%, Asian/Pacific – 3.4%, Asian – 3.1%, Average – 4.4%) and severity ratio (White – 6.7%, Black – 8.2%, Asian/Pacific – 0%, Asian – 10.3%, Average – 7.2%). The Hispanic/Latinx patients have higher positive ration than non-Hispanic/Latinx patients in both tasks (0.1% higher for hospitalization and 0.3% higher for severe outcomes). The consistency between actual statistics from the data and the model’s Shapley values demonstrates that MedML accurately captures the patterns from complex heterogeneous data with high reliability.

#### Model calibration

**Figure 20:**
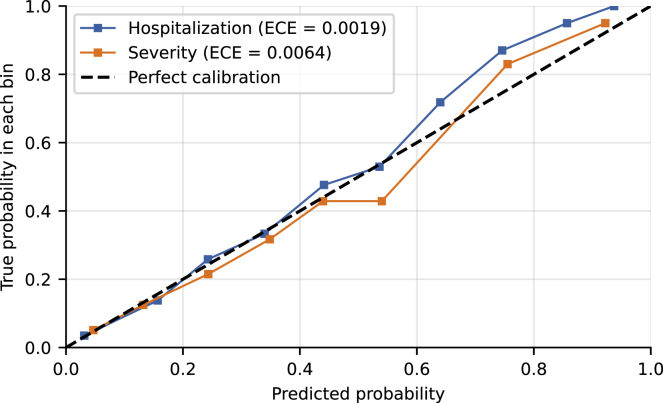
Model calibration curve and expected calibration error (ECE) for two tasks

The calibration plot of the model is shown in Figure 20.

In the calibration plot, the model prediction probabilities are divided into 10 bins representing the ranges of possible outcomes, e.g., [0–0.1), [0.1–0.2), etc. (i.e., the x axis). For each bin, the percentage of ground-truth positive samples is calculated (i.e., the y axis). The positive percentage of a perfectly calibrated model should correspond to the bin center. We also use the expected calibration error (ECE) to evaluate the calibration performance (Nixon et al., 2019). The ECE is calculated as:

$$ECE=\overset{B}{\underset{b=1}{\sum }}\frac{n_{b}}{N}\left|\bar{y_{b}}-\bar{\hat{y_{b}}}\right|$$

where B denotes the number of bins between 0 and 1, $n_{b}$ denotes the sample size in the b-th bin, $\bar{y_{b}}$ denotes the average of ground-truth labels in the bin, and $\bar{\hat{y_{b}}}$ denotes the average of predicted probability in the bin. The lower the ECE, the better the model is calibrated. The results in Figure 20 show that after the sigmoid regression calibration, the models for both tasks are well calibrated, which results in low ECE.

## Discussion

Compared to the best baseline model, MedML achieved a 3% higher area under the receiver operating characteristic (AUROC) and 4% higher area under the precision recall curve (AUPRC) on the hospitalization prediction task, and a 7% higher AUROC and 14% higher AUPRC on the severity prediction task. Additional analysis demonstrates that our hybridized approach built upon domain knowledge-based features can equal and even outperform the predictive performance of state-of-the-art data-driven methods using far fewer features. Detailed spatiotemporal analysis shows that MedML is generalizable in all nine national geographical regions and temporally across all consecutive pandemic stages. General feature analysis on our MedML can also explicitly show the inter-feature relationships and individual feature contributions for the final prediction results, which may help clinicians identify potential high-risk patients and related risk factors to inform individualized patient-care practices.

It should be noted that a previous version of MedML achieved even better performance, with a hospitalization task AUROC in the 80^th^ percentile. This was achieved by weighting a clinical diagnosis more heavily based on the total number of visits associated with that diagnosis code, e.g., 30 visits for cerebral palsy would result in a higher hospitalization prediction value than 3 visits. However, using such a model in clinical practice disadvantages, patients with barriers to accessing healthcare, thereby exacerbating the healthcare inequities faced by those individuals. Model performance must come secondary to model ethics; we hold ourselves to this principle.

MedML represents a new method of evaluating, automating, and informing existing clinical concepts and their relationships through a hybridized machine learning approach using a customized knowledge graph system. MedML is purpose-built on a framework of existing clinical knowledge using clinically intuitive concepts and relationships, on top of which it learns to identify new data-driven features which are both interpretable and quantifiable. In this way, the utility of the model stems not only from how it performs but also from what it provides in terms of feature importance. When MedML identifies a data-driven feature of high importance that is not within the original knowledge graph, that feature may represent one of three situations: 1) the feature was mistakenly omitted from the original graph and should be added, which serves to improve the underlying clinical knowledge graph; or 2) the feature is clinically irrelevant and represents either an error in the data or its engineering, which serves to improve data preprocessing and feature engineering; or 3) the feature may have true clinical relevance representing a previous unknown clinical relationship that merits further exploration.

To put it another way, our platform allows clinicians to visually compare knowledge-driven with data-driven features and quantify their relative levels of importance. While this comparison initially serves to improve the model, progressive iterations naturally lead to a validated set of features and feature importance values that can swing the pendulum from model improvement toward healthcare improvement by informing how a clinician perceives a patient’s individual risk factors based on the available data. A clinician may qualitatively understand that asthma and obesity are both predictive of COVID-19 severity, but they may not know which condition is quantitatively more predictive for particular patient given their unique combination of data elements and values, which is where MedML comes into play. The clinician informs the clinically relevant concepts, and MedML evaluates and quantifies those concepts while also exploring other concepts independently. In this way, the model both performs the automated predictive task and informs the physician’s internal predictive process in an iterative, synergistic platform whose impact reaches beyond its own performance to impact clinical practice itself. We hope that MedML serves as a bridge between clinicians, data engineers, and computer scientists to efficiently and powerfully augment the clinical decision-making process through intuitive knowledge representation, explainable construction, and powerful computation.

### Limitations of the study

This study is not without limitation. First, we did not have information on all pediatric cases in the United States, so study results may not generalize to all US pediatric population. Future works may include evaluating and applying this innovative method on different pediatric EHR datasets to assess its reproducibility among different cohorts. Our model also does not make additional improvements for the data misbalancing issues in both tasks, and this is intentional. Developing or utilizing techniques to balance the labels may further improve the prediction performance, but also risks exaggerating certain features and their importance. Similarly, we only use a simple but efficient metric, i.e., the propensity score, in the data-driven feature selection process. We do not explore other more complex feature selection algorithms since our target in this paper is to boost the prediction performance of machine learning models by fusing domain knowledge, rather than comparing performance differences brought about by feature selection and preprocessing techniques. In future application works, exploring more complex data preprocessing and feature selection algorithms may further improve MedML’s performance.

Another limitation is that the time related to each predictor was not included which could influence results. Future works may also include integrating recurrent neural networks to model the temporal patterns in EHR data. Furthermore, it is important to note that the factors identified in this study are correlative but not necessarily causative for COVID-19 among the pediatric population. Future studies are needed to adapt this model to other common conditions among hospitalized children; by comparing the resultant feature lists of several such conditions, we may begin to identify risk factors unique to COVID-19. The knowledge graph used for this task is meant to be representative of common relevant relationships; it is not meant to be exhaustive. Future development of the knowledge graph is needed to include all clinically relevant concepts and to connect those concepts to all appropriate data elements through detailed concept mappings across a wider breadth of relevant ontologies.

## STAR★Methods

### Key resources table

**Table:** 

REAGENT or RESOURCE	SOURCE	IDENTIFIER
**Deposited data**		

National COVID Cohort Collaborative	Haendel et al., 2021	https://ncats.nih.gov/n3c

**Software and algorithms**		

MedML	This Study	https://github.com/v1xerunt/MedML
Scikit-learn (version 0.23.2)	Pedregosa et al., 2011	https://scikit-learn.org/
XGBoost (version 1.0.2)	Chen and Guestrin, 2016	https://github.com/dmlc/xgboost
PyTorch (version 1.7.1)	Paszke et al., 2019	https://pytorch.org/

### Resource availability

#### Lead contact

Further information and requests for resources should be directed to and will be fulfilled by the lead contact, Jimeng Sun (jimeng@illinois.edu).

#### Materials availability

This study did not generate new unique reagents.

### Method details

#### Background

Current published studies on pediatric COVID-19 severity prediction primarily rely on statistical analysis to identify individual risk factors. Some also apply basic statistical models to predict severe outcomes such as death or cardiovascular support (Domínguez-Rodríguez et al., 2021; Martin et al., 2022; Nachega et al., 2022; Oliveira et al., 2022). Martin et al. (2022) analyzed the characteristics, outcomes, and risk factors of children with COVID-19 in the large multi-site N3C database. Nachega et al. (2022) analyzed the clinical outcomes and factors for children and adolescents hospitalized with COVID-19 in 6 countries in sub-Saharan Africa. Domínguez-Rodríguez et al. (2021) applied the naïve Bayesian model to predict COVID-19 severity within 350 children. Oliveira et al. (2022) applied the Fine and Gray competing-risks model to predict death outcomes for 10,017 pediatric patients using age, ethnicity, region, respiratory symptoms, and comorbidities features. To the best of our knowledge, there is no existing work which attempts these predictive tasks using a fusion of machine learning models with domain knowledge using heterogeneous, large-scale, multi-site EHR data.

For general COVID-19 related clinical tasks such as diagnosis (Feng et al., 2020; Zoabi et al., 2021), length of stay (Dan et al., 2020; Ma et al., 2021), or severity risk (Jamshidi et al., 2022; Wynants et al., 2020; Yan et al., 2020), many statistical or machine learning models have been proposed. Most of these cited works are purely data-driven, and none of them involve COVID-19 in children. Most are designed with limited features and are more likely to perform poorly on large heterogeneous datasets. For example, Ma et al. (2021) conducted length-of-stay prediction for COVID-19 patients from the HM Hospitals in Spain using a total of 66 lab test features for each patient, and Yan et al. (2020) conducted mortality prediction for COVID-19 patients from the Tongji Hospital in China using 74 lab test features. Most of these methods also have limited explainability due to their use of “black box” deep neural networks limiting their clinical utility.

The integration of domain knowledge into machine learning models is another ongoing research topic. Most existing works extract domain knowledge from medical literature or clinical concept hierarchies such as ICD codes. Lin et al. (2020) utilized a medical knowledge graph with medical entity alignment techniques to predict mortality risk for ICU patients. Choi et al. (2017) enriched their disease code embeddings with ICD-9 health information for diagnosis prediction. Despite their initial success in specific clinical applications, knowledge graphs in these works are extracted from only one clinical aspect, such as disease code ontology; however, different types of medical concepts, including lab tests, procedures, and disease codes, may have complex relationships. Bennett et al. (2021) trained multiple machine learning models on the N3C dataset, including gradient boosted tree and linear regression, to determine severity prediction for hospitalized adult patients with COVID-19 by selecting 64 input variables based on domain knowledge. Elliott et al. (2021) trained a logistic regression model on the UK Biobank COVID-19 dataset using 42 potential risk factors selected based on medical literature. In these works, domain knowledge is used only to limit the input feature size and does not guide the model learning process. Despite these current works, the problem of combining expert clinical knowledge with data-driven machine learning approaches to extract complex heterogeneous inter-feature relationships while achieving good predictive performance and interpretability remains an unsolved challenge.

#### Medical knowledge graph construction

Medical knowledge guides clinicians in making informed decisions for patients with COVID-19. Incorporating medical knowledge can guide the feature selection process among many medical concepts and enhance the performance of machine learning models. In this work, we construct a graph-based structure to represent such medical knowledge.

Concretely, we begin by constructing a heterogeneous COVID-19 medical knowledge graph. The nodes in the knowledge graph are medical concepts, and the edges are relationships between concepts. Our purpose is to utilize expert knowledge in clinical practice to learn enriched patient embeddings. Therefore, we build the graph based on both clinical practice from domain experts and risk factors in existing pediatric COVID-19 research (Bennett et al., 2021; Martin et al., 2022; Nachega et al., 2022; Oliveira et al., 2022). There are many well-established clinical knowledge bases from which clinical knowledge graphs have been created, such as SNOMED-CT. However, the relationships inherent within these systems are primarily ontological. To mimic the clinical hospitalization prediction thought process, we required another layer of abstraction on top of the ontological layer. This customized layer represents complex clinical concepts, (e.g., congenital cardiac malformations) and connects them to more discrete concepts (e.g., hypoxemia during time of illness) through an edge representing an “increases risk of” relationship. This conceptual hierarchy allows clinicians to more naturally map out their thought processes, and these clinically intuitive concepts are then connected to nodes representing discrete data elements using ICD-10, SNOMED-CT, and other processes specific to the N3C dataset task. The nodes in the knowledge graph have five types: *major condition*, *other condition*, *drug*, *measurement*, and *procedure*, which are derived from the reported major medical feature types in existing works. The primary condition nodes, for example, hypoxemia or respiratory distress, are diagnoses; they are the major reasons for hospitalization and underlie the need for ventilation or cardiovascular intervention for pediatric patients with COVID-19. The *drug*, *measurement*, and *procedure* nodes are medical concepts associated with other condition nodes. The relationship (i.e., edge) between two nodes is derived from clinical practice and medical literature. For example, we consider hypoxemia as a primary reason for hospitalization in children with COVID-19, which is a *major condition* node in our graph G. Chronic respiratory failure is one of the direct factors that increases the risk of hypoxemia from COVID-19 and is therefore classified as *other condition*. Chronic respiratory failure can also be related to severe cerebral palsy (which is also considered to be an *other condition* node) through several clinical relationships (e.g. kyphosis, scoliosis, upper airway obstruction, central apnea, chronic aspiration, etc.), so a directed edge pointing from cerebral palsy to chronic respiratory failure is created.

To this end, we can use this constructed knowledge graph to represent a patient’s clinical risk factors and map them to their EHR records. A patient will not have all the medical features in the knowledge graph, so we represent their historical records as $G_{p}\left(V_{p},E_{p}\right)$, where $G_{p}$ is a subgraph of G, $V_{p}$ and $E_{p}$ are subsets of the entire node set V and edge set E. We use an initialized dictionary $D\in R^{F\times E}$ to represent node embeddings of G, where F denotes the number of nodes (i.e., medical features in the graph) and E denotes the dimension of the embedding. To enrich the embedding, we also concatenate an extra dimension (i.e., column) to D, which indicates the historical existence (for conditions), frequency (for drugs and procedures) or the average value (for measurements) of a medical code in the patient’s entire visit history. For example, we append the frequency of central venous catheter as the extra dimension for central venous catheter node and add average blood pressure lab results as extra dimension for the blood pressure node. Finally, the embedding vector for node i in G is denoted as $v_{i}\in R^{E+1}$. Note that the extra dimension is not learnable during model optimization. When constructing the patient-level graph $G_{p}$, the initial node features are copied from the enriched dictionary. In this setting, different patients may have different graph structures and different node embeddings even for the same feature (frequency or average values might vary). This enables the model to learn personalized predictions for each patient in a flexible way.

The constructed knowledge graph consists of 65 medical features. The feature links in the knowledge graph can be found in the code repository and the features are listed below:

• Conditions (37): Asthma, Anxiety, Metabolic derangement, Obesity, Depression, Apnea, Dehydration, Failure to thrive, Respiratory distress, Atrial septal defect, Diabetes, Prematurity, Panic disorder, Post-traumatic stress disorder, Malignancy, Immunodeficiency, Malnutrition, Ventricular septal defect, Cerebral palsy, Renal injury, Congenital cardiac anomaly, Chronic kidney disease, Hypoxemia, Pulmonary hypertension, Heart failure, Chronic respiratory failure, Tetralogy of fallot, Congenital pulmonary anomaly, Acute heart failure, Marasmus, On ventilator, High altitude, Inborn error metabolism, Secondary infection, Congenital cystic adenomatoid malformation, Kwashiorkor, Gaucher disease.
• Drugs (15): Albuterol, Dexamethasone, Clonidine, Insulin, Tacrolimus, Methotrexate, Vincristine, Levocarnitine, Cyclophosphamide, Filgrastim, Doxorubicin, Daunorubicin, Sirolimus, Vinblastine, Bleomycin.
• Procedures (5): Tumor resection, Bone marrow biopsy, Central venous catheter, Gastrostomy tube placement, Cardiac catheterization.
• Measurements (8): BMI, Glucose, Creatinine, Weight, Spo2, GFR, A1c, Pao2.

#### Patient embedding learning using GAT

In traditional machine learning models, the input features are vectors, where each dimension indicates the numerical value (e.g., lab test result) or the existence of medical condition features. Common models cannot process the inter-relationships between features and are thus unable to utilize the hierarchical relationships such as the model representation. In MedML, we employ the graph attention networks (GAT) to incorporate the hierarchical relationships between medical features into the model. The basic premise is to learn node embeddings for each medical feature by aggregating information from its neighboring nodes. For example, since chronic respiratory failure and hypoxemia are closely related, this relationship will be learned and used to enrich their own node embeddings during the model learning process. The GAT model is a natural choice since it adaptively learns the attention score between the node and its neighbors and then aggregates the neighboring information in proportion to the score.

We use a two-layer GAT to learn the hierarchical relationships from the knowledge graph. Concretely, given a patient’s feature graph $G_{p}$, we apply the graph attention mechanism to calculate the node embedding $z_{i}\in R^{F_{z}}$ for node i based on the initial embedding $v_{i}$ and the information aggregated from its neighbors. $F_{z}$ denotes the dimension of the final node embeddings. Mathematically, given two adjacent nodes i and j, we calculate the attention weight between the two nodes as:

$$e_{ij}=\sigma \left(W_{a}\left(W_{z}v_{i}\|W_{z}v_{j}\right)\right)\text{,}$$

where $W_{z}\in R^{F_{z}\times \left(E+1\right)}$ denotes the weight matrix, $W_{a}\in R^{1\times 2F_{z}}$ denotes the attention computation matrix, (·||·) denotes the vector concatenation operation, and σ(⋅) is the non-linear activation function. Matching the original GAT model, we use a leaky rectified linear unit (LeakyReLU) as the activation function:

$$\sigma \left(x\right)=\;\left\{ \begin{array}{ll} x, & if\;x\geq 0,\\ 0.01x, & otherwise. \end{array}\right.$$

The attention score of all adjacent nodes is normalized using the SoftMax function:

$$a_{ij}=\text{softmax}\left(e_{ij}\right)=\frac{\exp\left(e_{ij}\right)}{\sum_{k=1}^{N_{i}}\exp\left(e_{ik}\right)}\text{,}$$

where $N_{i}$ denotes the number of neighbors of the node i. Each adjacent node j connected to the node i will have an attention score $a_{ij}$, indicating how much information will be aggregated to contribute to the update of node i. A higher attention score indicates the information from that adjacent node is more important. Finally, we obtain the updated embedding of node i by summing information from all adjacent nodes using attention scores as the weights:

$$z_{i}=\sum_{j=1}^{N_{i}}a_{ij}W_{z}v_{i}\text{.}$$

The updated embedding $z_{i}$ integrates the hierarchical information from all related nodes. Collectively, all node embeddings are stored in embedding matrix Z. To guide the learning process of node embeddings, we need supervision to optimize the weight matrix W and the initial embedding matrix D. After learning node embeddings, we obtain the graph embedding g with maxpooling over the node embedding Z and then feed the embedding into a multi-layer perceptron (MLP) for prediction:

g=Maxpool(Z),

$${\hat{y}}_{g}=MLP\left(g\right)\text{.}$$

The obtained graph embedding g summarizes the structural information from the knowledge graph. All model parameters are optimized with back-propagation using binary cross-entropy loss:

$$L=-y\;\log\left({\hat{y}}_{g}\right)-\left(1-y\right)\text{log}\left(1-{\hat{y}}_{g}\right)\text{.}$$

We use the back-propagation algorithm with the binary outcome labels y to optimize the graph neural network parameters. ${\hat{y}}_{g}$ here is only an auxiliary output and it’s not the final prediction score.

#### Data-driven feature selection

The clinical features used in the medical knowledge graph are extracted based on clinical practice and medical literature. In this section, we further enrich the knowledge-driven feature set with a purely data-driven method to discover new features that may not be obvious from previous research. By combining this data-driven feature selection with clinical knowledge graphs, our model can improve the final prediction accuracy and make it possible to identify new knowledge.

To select data-driven features, we consider the propensity score as a selection criterion, which is widely used to identify the significance of an indicator to the target outcome in the medical area (Haukoos and Lewis, 2015). We calculate the propensity score of each medical concept pScore based on the ratio of the positive and negative prevalence as:

$$pScore=\frac{f_{pos}/n_{pos}}{f_{neg}/n_{neg}}\;.$$

Here, $f_{pos}$ is the frequency of the medical concept in the positive patient cohort, $n_{pos}$ is the population size of the positive cohort, $f_{neg}$ is the frequency of the medical concepts in the negative patient cohort, and $n_{neg}\;$ is the population size of the negative cohort. A higher propensity score means a stronger prevalence of the medical concept in the positive cohort, and thus is likely to be a powerful indicator in clinical sense. Based on the propensity score, we select the top-K features, where K is a hyper-parameter. According to clinical research conventions, we use different encodings for different types of features. We use a binary encoding for condition features, where 1 denotes that the diagnosis code is recorded at least twice in the patient’s EHR data. For drug and procedure features, we use the number of encounters for each feature in the patients' EHR history. For measurement features, we use the average value of the features. These K features are finally concatenated as a vector, denoted by m. We conduct an ablation study on hyperparameter K in the experiment. We report the top 300 features with the highest propensity scores in the Tables S2 and S3.

#### Feature combining and prediction

The final step is to extract other medical features, combined with the learned patient graph embeddings and propensity score-based feature vectors, to make our predictions. In this section, we extract patient *static* demographic features, such as age, gender, race, etc., and represent them as multi-hot embedding vectors. We also consider exploiting evidential features from the *dynamic* patient visit sequences and transform them into numerical values, such as the average time gap between visits. For example, frequent and urgent patient hospital visits may reflect more complex or severe conditions and thus the patient is more likely to require hospitalization.

The visit features consists of the number of historical visit types, the number of historical visits and the average days between visits. The visit types include: Outpatient visit, Office visit, Interactive telemedicine service, Non-hospital institution visit, Telehealth, Emergency room visit, Inpatient visit, Parenteral and enteral nutrition supplier, Laboratory visit, Pharmacy visit, Ambulatory surgical center, Ambulatory clinic/center, Health examination, Observation room, Intensive care, Inpatient critical care facility, Home visit, Local education agency (lea), Mass immunization center, Managed care organization pharmacy, Outpatient skilled nursing facility visit, Case management visit, Nursing care coordination, Other/Unknown. The demographics are 32 categorical features and they are one-hot encoded into a binary vector. The demographic categories are:

• Gender (3): Male, Female, Other/Unknown
• Age (19): 0-18
• Race (7): White, Black, Asian, Asian or Pacific islander, Multiple races, Other Pacific islander, Other/Unknown
• Ethnicity (3): Hispanic or Latino, Not Hispanic or Latino, Other/Unknown

The static demographical features and the dynamic visit features are eventually concatenated, denoted as s. The final feature input into the predictive model comprises patient graph embedding g, propensity score-based feature vector m, and demographics and visit feature vector s. We use the gradient-boosted ensemble of decision trees (GBDT) model to perform the final prediction,

$$\hat{y}=GBDT\left(\left[g,\;m,\;s\right]\right)\text{.}$$

All model outputs are calibrated using Sigmoid regression calibration (i.e., Platt scaling, (Platt, 1999)), which fits a parametrized logistic regression model to minimize the deviation between the predicted probability and ground-truth labels.

### Quantification and statistical analysis

All statistics of the dataset and patient cohorts can be found in Tables 1 and 2 in the results section. Baseline models, software and codes, hyperparameter and dataset split settings can be found in the baselines and experiment settings section. All quantification performances are shown in the model performance analysis section. The standard deviations of performances are calculated by training the models 5 times using different random seeds. We conduct the Student’s *t* test to evaluate the significance of the performance differences. The results show that the performance differences between MedML and the best baseline models are significant (p <0.001).

## Consortia

Christopher G Chute, Melissa A Haendel, Davera Gabriel, N3C Consortium.

## Data Availability

This paper analyzes existing, publicly available N3C data. These accession URL for the N3C dataset is listed in the key resources table. Source code and tutorials for implementing the MedML model are publicly available online at https://github.com/v1xerunt/MedML. Any additional information required to reanalyze the data reported in this paper is available from the lead contact upon request.

## Supplemental information

Document S1. Table S1

Table S2. Top 300 features with highest propensity scores in the hospitalization prediction task, related to STAR Methods

Table S3. Top 300 features with highest propensity scores in the severity prediction task, related to STAR Methods
