# Supplementary material for: MedML: Fusing medical knowledge and machine learning models for early pediatric COVID-19 hospitalization and severity prediction
Source: iScience. 2022 Aug 17;25(9):104970. doi: 10.1016/j.isci.2022.104970 (PMC9384332; doi:10.1016/j.isci.2022.104970)
Supplement: Document S1. Table S1 [file mmc1.pdf]

iScience, Volume *25*

Supplemental information

MedML: Fusing medical knowledge and machine learning models for early pediatric COVID-19 hospitalization and severity prediction

Junyi Gao, Chaoqi Yang, Joerg Heintz, Scott Barrows, Elise Albers, Mary Stapel, Sara Warfield, Adam Cross, Jimeng Sun, and on behalf of the N3C consortium

Table S1 Notations used in this study, Related to STAR Methods.

Notation	Concept
$\boldsymbol{G}(\boldsymbol{\mathcal{V}}, \boldsymbol{\mathcal{E}})$	Knowledge graph G with node set $\mathcal{V}$ and edge set $\mathcal{E}$
$\boldsymbol{v}, \boldsymbol{e}$	A single node or edge in the knowledge graph
$\boldsymbol{i}, \boldsymbol{j}$	Node indices in the graph
$\boldsymbol{p}$	Patient EHR data vector
$\boldsymbol{y_h}, \boldsymbol{y_s}$	Hospitalization risk and severity prediction binary labels
$\boldsymbol{G_p}(\boldsymbol{\mathcal{V}_p}, \boldsymbol{\mathcal{E}_p})$	Knowledge graph for a single patient p, which is a subgraph of G
$\boldsymbol{D} \in \mathbb{R}^{\boldsymbol{F} \times \boldsymbol{E}}$	Initial graph node embedding dictionary
$\boldsymbol{F}$	Number of nodes in the graph $F = \lvert\boldsymbol{\mathcal{V}}\rvert$
$\boldsymbol{E}$	Embedding dimension
$\boldsymbol{v_i} \in \mathbb{R}^{\boldsymbol{E}+\boldsymbol{1}}$	Augmented embedding vector for node i
$\boldsymbol{W_z} \in \mathbb{R}^{\boldsymbol{F_z} \times (\boldsymbol{E}+\boldsymbol{1})}$	Parameter matrix in Graph attention network (GAT)
$\boldsymbol{W_a} \in \mathbb{R}^{\boldsymbol{1} \times \boldsymbol{2F_z}}$	Parameter matrix in GAT
$\boldsymbol{F_z}$	Final node embedding dimension
$\boldsymbol{a_{ij}}$	Attention score
$\boldsymbol{z_i}, \boldsymbol{Z}$	Final node embedding matrix
$\boldsymbol{g}$	Graph embedding vector
$\boldsymbol{\hat{y}_g}$	Predicted logit in binary classification
$\boldsymbol{m}$	Data driven feature vector
$\boldsymbol{s}$	Demographics and visit feature vector
